# Assessment of sub-clinical acute cellular rejection after heart transplantation: Comparison of cardiac magnetic resonance imaging and endomyocardial biopsy

**DOI:** 10.1186/1532-429X-16-S1-P276

**Published:** 2014-01-16

**Authors:** Matthias Gutberlet, Christian Krieghoff, Markus J Barten, Lysann Hildebrand, Matthias Grothoff, Lukas Lehmkuhl, Christian Luecke, Claudia Andres, Friedrich W Mohr

**Affiliations:** 1 Diagnostic and Interventional Radiology, University Leipzig - Heart Center, Leipzig, Germany; 2 Cardiac Surgery, University Leipzig - Heart Center, Leipzig, Germany

## Background

To compare the diagnostic value of multi-sequential cardiac magnetic resonance imaging (CMR) with endomyocardial biopsy (EMB) as the standard of reference for sub-clinical cardiac allograft rejection.

## Methods

On-hundred-forty-six CMR examinations in 73 Patients (mean age 53 ± 12 years, 58 male) were performed using a 1.5 Tesla MR scanner and compared to EMB results. A multi-sequential protocol including a T2-weighted STIR (short tau inversion recovery) sequence for calculation of the edema ratio (ER), a T1-weighted spin-echo sequence for the assessment of the global relative enhancement (gRE) as well as inversion-recovery sequences to visualize late gadolinium enhancement (LGE), with the same cut-off values for ER (≥2) and gRE (≥4.5) as for myocarditis was used. The presence of LGE was assessed qualitatively only. A histological grade ≥1B was considered as relevant rejection in which all patients received anti-inflammatory medical treatment.

## Results

One-hundred-twenty-seven (127/146 = 87%) EMBs demonstrated with no or mild signs of rejection (grades ≤1A) and 19/146 (13%) with a relevant rejection (grade ≥1B). Sensitivity, specificity, positive predictive (PPV) and negative predictive value (NPV) for rejection grade 1B or higher were as follows: ER: 63%, 78%, 30% and 93%; gRE: 63%, 70%, 24% and 93%; LGE: 68%, 36%, 13% and 87%; with the combination of ER and gRE with at least 1 out of 2 positive: 84%, 57%, 23% and 96%. A receiver operator characteristic analysis revealed an area under the curve of 0.724 for ER and 0.659 for gRE.

## Conclusions

CMR parameters for myocarditis were also useful to detect sub-clinical acute cellular rejection after heart transplantation. Comparable results to myocarditis could be achieved, with a combination of parameters.

## Funding

No funding.

